# Supplementary material for: Long-term health effects perceived by snakebite patients in rural Sri Lanka: A cohort study
Source: PLoS Negl Trop Dis. 2022 Sep 1;16(9):e0010723. doi: 10.1371/journal.pntd.0010723 (PMC9473613; doi:10.1371/journal.pntd.0010723)

**S1 Table.** Descriptions of the patients with musculoskeletal sequelae following snakebite and their impact.

| **Age**  **(years)** | **Sex** | **Occupation** | **Snake** | **Bite site** | **Local effects during the acute stage** | **Interventions during the acute stage** | **Current musculoskeletal problem** | **Impact on daily**  **activities according**  **to patient’s**  **perception** |  |
| --- | --- | --- | --- | --- | --- | --- | --- | --- | --- |
| **Group I** | | | | | | | | | |
| 20 | Male | Farmer | Russell’s viper | Dorsum of the left foot | Pain,  swelling,  secondary infection at the bite site and  cellulitis of the left foot. | Antivenom,  IV antibiotics,  wound debridement. Physiotherapy not done. | Slight dorsiflexion of the left big toe at rest due to a contracture. No restriction of movements. | Not affected on walking  or other activities. |  |
| 46 | Female | Farmer | Russell’s viper | Left index finger | Pain, swelling, and secondary infection at the bite site | Antivenom, IV antibiotics, Not undergone wound care in an allopathic center post-discharge and had taken native treatment. | Contracture formation at the distal inter-phalangeal joint. Restricted extension of the distal interphalangeal joint. | Patient is right  Handed, hence the effect  is negligible. |  |
| 63 | Female | Farmer | Merrem’s Hump-nosed viper | Right small finger | Pain, swelling, blistering, bluish discolouration, local tissue necrosis, and swelling of the right forearm | Fasciotomy of right forearm, oral and IV antibiotics, amputation of the right small finger at the proximal interphalangeal joint.  Physiotherapy not done. | Loss of distal part of the right small finger.  Fasciotomy scar involving the right forearm. No contracture formation. | Grip not affected and the movement of the wrist  joint is not affected hence the effect is negligible. |  |
| 26 | Female | Housewife | Merrem’s Hump-nosed viper | Left ring finger | Pain, swelling, regional lymphadenopathy, blistering, bluish discolouration of the bite site, and local tissue necrosis | Oral antibiotics. No surgical interventions and physiotherapy not done. | Contracture formation at the palmar aspect of the left ring finger causing permanently flexed distal phalanx. | The grip is not affected. The patient is right-handed. Cosmetic concerns with not being able to wear the wedding ring. |  |
| **Group II** | | | | | | | | |  |
| 27 | Male | Manual laborer | Merrem’s Hump-nosed viper | Dorsum of the right hand | Pain, swelling extending to the elbow. | Oral antibiotics and IV antibiotics.  Fasciotomy extending from right dorsum of the hand to forearm.  No physiotherapy. | Massive fasciotomy scar extending from the dorsum of the right hand to elbow. | No functional impairment of the upper limb. Cosmetic concerns of a big scar. |  |
| 33 | Female | Farmer | Merrem’s Hump-nosed viper | Right thumb | Pain, swelling, blister formation, bluish discolouration, and necrosis | Oral antibiotics and IV antibiotics.  Fasciotomy of the dorsum of right thumb | Tissue loss in the plantar aspect of the right thumb and fasciotomy scar in the dorsum. | No disabilities. |  |
| 61 | Female | Farmer | Merrem’s Hump-nosed viper | Right ring finger | Pain, swelling, bluish discolouration, Necrosis | IV antibiotics, wound debridement done. | Small tissue loss with the scar at the bite site. | No disabilities. |  |
| 53 | Female | Farmer | Merrem’s Hump-nosed viper | Left ring finger | Pain, swelling | IV antibiotics | Small scar at the bite site. | No disabilities |  |
| 68 | Female | Housewife | Merrem’s Hump-nosed viper | Right ring finger | Pain, swelling, blistering, bluish discolouration. | IV antibiotics,  wound debridement done. | Small tissue loss with bite site scar. | No disabilities. |  |
| 43 | Male | Retired navy officer | Merrem’s Hump-nosed viper | Right index finger | Pain, swelling, blistering, bluish discolouration, necrosis | IV antibiotics, wound debridement, fasciotomy over the right index finger. | Fasciotomy scar over the right index finger | No disability. |  |
| 27 | Male | Farmer | Merrem’s Hump-nosed viper | Left first toe | Pain, swelling, bluish discolouration | IV antibiotics | Small scar at the bite site | No disabilities |  |
| 53 | Male | Farmer | Merrem’s Hump-nosed viper | Right small toe | Pain, swelling | IV antibiotics | Small tissue loss with bite site scar. | No disabilities |  |
| 58 | Male | Farmer | Merrem’s Hump-nosed viper | Left third toe | Pain, swelling, bluish discolouration | IV antibiotics | Contracture formation at the plantar aspect of the left third toe.* | No disabilities |  |
| 49 | Female | Clerk | Russell’s viper | Right middle finger | Pain, swelling, bluish discolouration , Necrosis | Antivenom,  IV antibiotics | Small tissue loss with bite site scar | No disabilities |  |
| 41 | Female | Housewife | Russell’s viper | Lateral aspect of the right lower leg | Pain, swelling,  Bluish discolouration. The wound appeared two weeks after the bite. | Antivenom, oral antibiotics,  wound Debridement. | Bite site scar. | No disabilities. |  |
| 20 | Female | Housewife | Russell’s viper | Left big toe | Pain, Swelling, Bluish discolouration | Antivenom, oral antibiotics. | Small scar at the medial aspect of the left big toe | No disabilities |  |


Supplement: S1 Table — (DOCX) [file pntd.0010723.s001.docx]
