# Supplementary material for: Long-term health effects perceived by snakebite patients in rural Sri Lanka: A cohort study
Source: PLoS Negl Trop Dis. 2022 Sep 1;16(9):e0010723. doi: 10.1371/journal.pntd.0010723 (PMC9473613; doi:10.1371/journal.pntd.0010723)
Supplement: S2 Table — (DOCX) [file pntd.0010723.s002.docx]

**S2 Table.** Local effects persistent in reviewed patients at the review.

|  | **Russell’s viper**  **(n_1_=93)**  **(n_2_=67)** | **Merrem’s Hump-nosed viper**  **(n_1_=49)**  **(n_2_=49)** | **Indian krait**  **(n_1_=12)**  **(n_2_=5)** | **Common cobra**  **(n_1_=0)**  **(n_2_=2)** | **Identified non/mildly venomous snakes**  **(n_1_=15)**  **(n_2_=19)** | **Snake unidentified**  **(n_1_=30)**  **(n_2_=26)** | **All** |
| --- | --- | --- | --- | --- | --- | --- | --- |
| **On and off mild pain: n, (%)** | | | | | | | |
| Phase I | 11(12) | 3(6) | 0 | 0 | 0 | 0 | 14 |
| Phase II | 7(10) | 3(6) | 1(20) | 0 | 0 | 3(12) | 14 |
| **On and off tingling sensation: n, (%)** | | | | | | | |
| Phase I | 6(6) | 2(4) | 0 | 0 | 0 | 0 | 8 |
| Phase II | 2(3) | 1(2) | 0 | 0 | 0 | 0 | 3 |
| **On and off numbness: n, (%)** | | | | | | | |
| Phase I | 9(10) | 0 | 0 | 0 | 0 | 0 | 9 |
| Phase II | 2(3) | 1(2) | 0 | 0 | 0 | 0 | 3 |
| **On and off local swelling: n, (%)** | | | | | | | |
| Phase I | 6(6) | 0 | 0 | 0 | 0 | 0 | 6 |
| Phase II | 3(4) | 0 | 0 | 0 | 0 | 1(4) | 4 |

n_1_ – number of reviewed patients in phase I, n_2_ – number of reviewed patients in phase II
