# Supplementary material for: Long-term health effects perceived by snakebite patients in rural Sri Lanka: A cohort study
Source: PLoS Negl Trop Dis. 2022 Sep 1;16(9):e0010723. doi: 10.1371/journal.pntd.0010723 (PMC9473613; doi:10.1371/journal.pntd.0010723)
Supplement: Attachment — Submitted filename: Response to reviewers.docx [file pntd.0010723.s003.docx]

ABSTRACT

Reviewer #2:
•       There are many methodological limitations of this study, and none are mentioned in the abstract. For example, the participation rates were relatively low, the outcome is based on “perception” which may be subject to information bias, and no relative measures of association are reported in the abstract.

**However, as we have stated in the title and the aims, we are intentionally describing ‘perceived health effects’, rather then objective medical effects. It is therefore only a minor limitation of this study. The importance of documenting ‘perceived effects’ is not to miss any effects that are likely to be missed by the western bio-medical model of health (state of health is defined purely in the absence of illness according to disease definitions that are in use). Such a limitation has been discussed in the main body of the paper, but we do not believe that needs to be covered in the abstract.**

Reviewer #3:
The description is unclear. was group1 recruited 2013-2014 or was this the date of follow-up, i.e. the recruitment was 4 years earlier. same question regarding group 2. It becomes clear only after reading the full text.

**To make this clearer, we amended the sentence as “Two groups of snakebite patients (>18y) from the Anuradhapura snakebite cohort were reviewed: Group I had a snakebite during August 2013-October 2014 and was reviewed after 4 years, and group II had a snakebite during May 2017-August 2018, and were reviewed after one year.”**

METHODS

Reviewer #1:
The methodology is generally correct, adapted to the purposes of the study which is mainly de-scriptive.

**Thank you.**

Reviewer #2:
•       Lines 142-151: It is unclear whether the patients in group I had a different authentication method than patients in group II. If so, this can result in both selection bias and information bias and must be acknowledged and addressed by the authors.

**We thank the reviewer for these comments. We have already stated the exact authentication carried out for the two groups in the methods. As per the reviewer’s suggestion, we have now included a much more extensive discussion of this as a limitation.**

•       The selection of patients in 2013/2014 vs. 2017/2018 can result in selection bias. I understand that the one year vs. four year follow-up is evaluated. However, differential selection of participants based on different years of study entry may introduce factors in one group (vs. the other) that are not present or which may vary with the other group. For example, did treatment in 2013/2014 differ between 2017/2018? Did access to care differ during those time periods?

**Thank you for raising this issue. There was a 3-year gap between the two groups, and the patient management guidelines, the treatment or access to healthcare were not changed during this period. The aim of this study was “to describe the long-term effects of snakebite among patients followed up at one and four years post-bite from a snakebite cohort in rural Sri Lanka, and to investigate the factors associated the development of long-term effects of snakebite”. However, we have compared the prevalence of certain health effects between the two groups, so we have provided a detailed section on limitations, admitting the selection bias likely to affect the conclusions.**

•       Lines 200-202: It appears as though you are conflating multicollinearity with confounding. These are two different concepts. Multicollinearity occurs when the covariates are highly correlated with each other (potentially resulting in unstable risk estimates). Confounding factors are related independently to the exposure of interest and the outcome of interest.

**We have amended the sentence as “The multiple logistic regression model was developed with a forward selection approach, and multicollinearity was considered in removing potentially interrelated independent variables.”**

Reviewer #3:
Generally, it is fine to perform a logistic regression.  I wonder why the authors used separate logistic models for Group 1 and 2. It would be useful to have a joint model, with the group (resp. the time after snake bite) as a covariable. This would also give an adjusted estimate to show a possible difference between one and four years after snake bite. The Fisher test at the beginning of the results section would then become redundant.   
A dichotomization of continuous covariables (here: age) is generally not recommended (see. for example, Patrick Royston, Douglas G Altman, Willi Sauerbrei. Dichotomizing continuous predictors in multiple regression: a bad idea. Stat Med . 2006 Jan 15;25(1):127-41. )

**We thank the reviewer for this valuable comment. The logistic regression was done again with the two groups combined, with the time after snakebite as a covariable. We agree with the reviewer that dichrotomisation of age is not ideal. However, we used that for convenience of the analysis based on the fact that similar to many developing countries worldwide, Sri Lanka defines the elderly population as those who have completed sixty years of age and above.**

RESULTS

Reviewer #1:
The described situation is accurately reported, which is an essential starting point for future dis-cussions. It confirms other studies, particularly in Africa, which had shown, on the one hand the low frequency of physical sequelae and on the other hand the importance of psychological disorders following snakebites.

**We thank the reviewer for the comments.**

Reviewer #2:
•       Lines 208-209: the participation rates were low. This should be acknowledged in the discussion section [you do this but do not discuss the statistical implications of your results]. Furthermore, Table 1 should separate patients reviewed by not reviewed rather than by reviewed compared to all. This results in misleading population characteristics. For example, 47% of reviewed patients in group I were bitten by a Russell’s viper and 34% of all patients were bitten by a Russell’s viper. However (when I did the math), 28% of non-reviewed study subjects were bitten by a Russell’s viper compared to 47% of those reviewed. This is the more appropriate way to present the study. My strong recommendation is to re-create Table 1.

**We amended the table 1 as suggested by the reviewer and discussed the issues under limitations.**

•       Was/is it possible to evaluate the same group at one year of follow-up and then two and so on? This would give you better control of potential confounding factors.

**We thank the reviewer for the suggestion. Although we initially planned to follow-up the patients in group II after 2 years as well, it was not possible due to the COVID restrictions.**

•       If the focus of the study is to compare symptoms and adverse health-related issues four years vs. one year after snakebite, why didn’t you conduct that very analysis? Supplementary table 2 shows the regression analyses but they are within group and not between group (which would be the most informative). Without this type of analysis, you only have descriptive within group data that does not directly address the scope of your research question.

**The aim of this study was “to describe the long-term effects in snakebite patients followed up at one and four years post-bite from a snakebite cohort in rural Sri Lanka and to investigate factors associated with patients developing long-term effects of snakebite.” and not necessarily to compare the effects after 1 and 4 years. Therefore, our analysis was mainly descriptive and aimed to explain the factors associated with the reported medically-unexplained in each group, we conducted the logistic regression. As per the suggestion by reviewer #3, we have combined the group I and II and re-did the logistic regression.**

Reviewer #3:
A major issue is the question whether the reviewed patients can be considered as representative sample of all patients. The numbers in table 1 show that there might be some doubt. A higher percentage received antivenom, and of these, a higher percentage showed acute adverse reactions. This is only briefly discussed on lines 393-397. It is unclear, however, what impact that could have on the result.

**As per the suggestion by the reviewer #2, table 1 now shows the information of non-reviewed vs reviewed patients in each group.**

**We have now explicitly discussed the selection bias associated with this under the limitations.**

Table 1, last row:      The percentage should be relative to those which received antivenom

**Corrected**

Supplemental tables:    since the logistic regression analysis is described in detail in the methods section, I wonder Why the results are hidden in the supplement.

**We have now presented the logistic regression under the results and not as a supplemental table.**

It is not appropriate to give OR estimates, p-values and confidence intervals with four digits after the decimal point. two is more than enough.

**Corrected.**

Supp. table 3 looks sophisticated, however not too useful. It is not clear how “Type of snake”, apparently the most relevant predictor, has been formalized and which snake is associated with a higher risk. Z-value and p-value can be derived from each other, thus one of both is redundant. The equations with the estimated regression coefficients are not useful either.

**We re-did the logistic regression by combining the two groups, as suggested, and type of snake is not included in the final model now.**

**We have removed the z-value from the table, but would like to retain the the equations and estimated regression coefficients, for a better comprehension for the readers.**

CONCLUSIONS

Reviewer #2:
There are many methodological limitations of this study. For example, the participation rates were relatively low, the outcome is based on “perception” which may be subject to information bias, and no relative measures of association are reported in the abstract.

**We have included these limitations in the discussion and conclusions.**

DISCUSSION

Reviewer #3:
The discussion is rather lengthy and should be streamlined.However, the limitations of the study and possible directions of biased results need more emphases

**We have amended the discussion giving more emphasis to the limitations.**

SUMMARY AND GENERAL COMMENTS

Reviewer #1:
The manuscript by Waiddyanatha et al. “Long-term health effects perceived by snakebite patients in rural 2 Sri Lanka: a cohort Study” concerns a crucial issue, with strong impacts on daily activities and livelihood of concerned people, and on the economy of large rural areas, and yet very poorly addressed.
The authors report a high proportion (25 to 50%) of people bitten by a snake complaining of symptoms, which were in the minority physical (amputations, functional deficits, musculoskeletal disorders, unsightly sequelae). However, most disorders were psychological in nature – or physical but attributed to biting without clear evidence – and may be culturally dependent. Their real impact on their daily lives is difficult to assess although probably not critical.
My major concern – partly mentioned by the authors – concerns the poor representativeness of the sample of patients interviewed by the authors, both geographically and statistically (low percentage of examined patients from the cohorts).
The authors could suggest modifying the protocol to overcome these flaws (in addition to recommend surveys in other regions to corroborate their results) and make it possible to verify the hypothesis regarding the possible role of cultural factors in the sequelae not directly linked to the action of the venom. It could be useful to include patients who have suffered from other severe or shocking pathologies to look for this type of sequelae. While waiting for such investigations, the authors could seek other studies already carried out on this subject regarding pathologies other than snakebite envenomation.

**We thank the reviewer for the valuable comments and suggessions. We have now explicitly discussed the limitations, including the selection bias. We have taken multiple steps to increase the number of participants (sending letters, telephone calls and home visits), practical in this sort of developing setting. The way forward in understanding the ‘medically unexplained symptoms’ is to conduct qualitative studies further exporing the possible socio-cultural elements.**

Reviewer #2:
Discussion and Overall Methodological Comments:
•       You did not perform the necessary regression analyses to compare four year vs. one year differences. Furthermore, such analyses would need to account for potentially relevant confounding factors. For example, if you were to compare a self-reported health issue at four years vs. one year, you would need to statistically adjust for relevant factors, such as the severity and location of the bite, timing of treatment, etc. A between group table that shows logistic regression data should be included.

**We have re-done the logistic regression combining the groups and including time since snakebite as a covariable**.

•       Also, what about effect modification? For example, did systemic envenoming or antivenom modify the reported associations? This is very important and such stratified analyses would be informative.

**We are thankful to the reviewer for pointing this out. We’ve checked for potential effect modifiers, and a substantial effect modification could not be elicited. Nonetheless, there may be potential pitfalls in the over-analysis of self-reported quantitative data generated from this kind of study, leading to interpretations without the support of adequate socio-cultural cross-validation. This should be investigated through qualitative studies such as focus-group discussions and in-depth interviews of the patients with medically unexplained symptoms. As detailed in the discussion, for the further understanding of the factors related to ‘medically unexplained symptoms’, it will be pivotal to explore this with multiple approaches, including qualitative studies.**

•       Very few methodological and analytical limitations are discussed in your paper. You need to acknowledge and address these important issues. The word “bias” does not even show up in your paper even though your study is subject to information bias, recall bias, and selection bias, among other types of biases (e.g., diagnostic bias). In addition, you do not discuss confounding even though this is a significant concern as I mentioned above.

**We have explicitly discussed the limitations now.**
