# Supplementary material for: Long-term health effects perceived by snakebite patients in rural Sri Lanka: A cohort study
Source: PLoS Negl Trop Dis. 2022 Sep 1;16(9):e0010723. doi: 10.1371/journal.pntd.0010723 (PMC9473613; doi:10.1371/journal.pntd.0010723)
Supplement: Attachment — Submitted filename: review_PNTD-D-22-00201.docx [file pntd.0010723.s004.docx]

My responses to the author’s responses are highlighted in yellow

ABSTRACT

Reviewer #3:
The description is unclear. was group1 recruited 2013-2014 or was this the date of follow-up, i.e. the recruitment was 4 years earlier. same question regarding group 2. It becomes clear only after reading the full text.

**To make this clearer, we amended the sentence as “Two groups of snakebite patients (>18y) from the Anuradhapura snakebite cohort were reviewed: Group I had a snakebite during August 2013-October 2014 and was reviewed after 4 years, and group II had a snakebite during May 2017-August 2018, and were reviewed after one year.”**

ok

METHODS

Reviewer #3:
Generally, it is fine to perform a logistic regression.  I wonder why the authors used separate logistic models for Group 1 and 2. It would be useful to have a joint model, with the group (resp. the time after snake bite) as a covariable. This would also give an adjusted estimate to show a possible difference between one and four years after snake bite. The Fisher test at the beginning of the results section would then become redundant.   
A dichotomization of continuous covariables (here: age) is generally not recommended (see. for example, Patrick Royston, Douglas G Altman, Willi Sauerbrei. Dichotomizing continuous predictors in multiple regression: a bad idea. Stat Med . 2006 Jan 15;25(1):127-41. )

**We thank the reviewer for this valuable comment. The logistic regression was done again with the two groups combined, with the time after snakebite as a covariable. We agree with the reviewer that dichrotomisation of age is not ideal. However, we used that for convenience of the analysis based on the fact that similar to many developing countries worldwide, Sri Lanka defines the elderly population as those who have completed sixty years of age and above.**

I find this not completely convincing. Anyway, age does not appear to have a relevant effect, so this may be acceptable.

RESULTS

Reviewer #3:
A major issue is the question whether the reviewed patients can be considered as representative sample of all patients. The numbers in table 1 show that there might be some doubt. A higher percentage received antivenom, and of these, a higher percentage showed acute adverse reactions. This is only briefly discussed on lines 393-397. It is unclear, however, what impact that could have on the result.

**As per the suggestion by the reviewer #2, table 1 now shows the information of non-reviewed vs reviewed patients in each group.**

**We have now explicitly discussed the selection bias associated with this under the limitations.**

ok

Table 1, last row:      The percentage should be relative to those which received antivenom

**Corrected**

ok

Supplemental tables:    since the logistic regression analysis is described in detail in the methods section, I wonder Why the results are hidden in the supplement.

**We have now presented the logistic regression under the results and not as a supplemental table.**

ok

It is not appropriate to give OR estimates, p-values and confidence intervals with four digits after the decimal point. two is more than enough.

**Corrected.**

**This seems to be incorrect. Most tables still report 4 digits**

Supp. table 3 looks sophisticated, however not too useful. It is not clear how “Type of snake”, apparently the most relevant predictor, has been formalized and which snake is associated with a higher risk. Z-value and p-value can be derived from each other, thus one of both is redundant. The equations with the estimated regression coefficients are not useful either.

**We re-did the logistic regression by combining the two groups, as suggested, and type of snake is not included in the final model now.**

**We have removed the z-value from the table, but would like to retain the the equations and estimated regression coefficients, for a better comprehension for the readers.**

I am not happy with tables 5 and 6, and with the model selection procedure. The authors write that they used a forward selection approach. This is apparently not the case, since age and sex, two almost irrelevant variables, were listed in the first model. The modelling sequence listed in table 6 cannot be the result of forward selection. A better description is needed here.
