# Supplementary material for: Long-term health effects perceived by snakebite patients in rural Sri Lanka: A cohort study
Source: PLoS Negl Trop Dis. 2022 Sep 1;16(9):e0010723. doi: 10.1371/journal.pntd.0010723 (PMC9473613; doi:10.1371/journal.pntd.0010723)
Supplement: Attachment — Submitted filename: Response to reviewer 15JUL2022.docx [file pntd.0010723.s005.docx]

We have responded only to the two comments by us that the Reviewer #3 was not satisfied with. The reviewer has accepted our other responses.

1.
Reviewer at the 1^st^ round of comments:
It is not appropriate to give OR estimates, p-values and confidence intervals with four digits after the decimal point. two is more than enough.

Our response: Corrected.

Reviewer at the 2^nd^ round of comments:
This seems to be incorrect. Most tables still report 4 digits

**Our response: We apologise for the mistake. The tables are now reporting the p values, OR estimates with confidence intervals only to the two decimal points.**

2.

Reviewer at the 1^st^ round of comments:
Supp. table 3 looks sophisticated, however not too useful. It is not clear how “Type of snake”, apparently the most relevant predictor, has been formalized and which snake is associated with a higher risk. Z-value and p-value can be derived from each other, thus one of both is redundant. The equations with the estimated regression coefficients are not useful either.

Our response: We re-did the logistic regression by combining the two groups, as suggested, and type of snake is not included in the final model now.

We have removed the z-value from the table, but would like to retain the the equations and estimated regression coefficients, for a better comprehension for the readers.

Reviewer at the 2^nd^ round of comments:

I am not happy with tables 5 and 6, and with the model selection procedure. The authors write that they used a forward selection approach. This is apparently not the case, since age and sex, two almost irrelevant variables, were listed in the first model. The modelling sequence listed in table 6 cannot be the result of forward selection. A better description is needed here.

**Our response: In the previous approach, multiple logistic regression analysis was done following a purposeful selection of the predictor variables, having medically unexplained symptoms as the dependent variable. Age and gender variables were purposefully retained in the model, considering the biological plausibility. Other predictor variables for the regression model were selected based on the statistical significance of bivariate analysis. These selected variables were considered in the initial model with age and gender, in a forward selection manner, to achieve the final model.**

**Here we agree with the reviewer’s suggestion regarding our original approach, and we have now performed the binary logistic regression analysis with a complete forward selection method. We revised the data analysis and results sections with more details, and revised table 6 to include variables selected for the final model.**
